# Updating global urbanization projections under the Shared Socioeconomic Pathways

**DOI:** 10.1038/s41597-022-01209-5

**Published:** 2022-03-31

**Authors:** Shiyin Chen, Qingxu Huang, Raya Muttarak, Jiayi Fang, Tao Liu, Chunyang He, Ziwen Liu, Lei Zhu

**Affiliations:** 1 State Key Laboratory of Earth Surface Processes and Resource Ecology, Beijing Normal University, Beijing, 100875 China; 2 School of Natural Resources, Faculty of Geographical Science, Beijing Normal University, Beijing, 100875 China; 3 International Institute for Applied Systems Analysis (IIASA), Laxenburg, Austria; 4 Wittgenstein Centre for Demography and Global Human Capital (IIASA, OeAW, University of Vienna), Vienna, Austria; 5 Department of Statistical Sciences, University of Bologna, Bologna, Italy; 6 Key Laboratory of Geographic Information Science, Ministry of Education, School of Geographic Sciences, East China Normal University, Shanghai, 200241 China; 7 College of Urban and Environmental Sciences, Peking University, Beijing, China; 8 Ministry of Education Key Laboratory for Earth System modeling, Department of Earth System Science, Tsinghua University, Beijing, 100084 China

**Keywords:** Socioeconomic scenarios, Sustainability, Climate-change adaptation, Projection and prediction, Climate-change policy

## Abstract

Urbanization level is an important indicator of socioeconomic development, and projecting its dynamics is fundamental for studies related to global socioeconomic and climate change. This paper aims to update the projections of global urbanization from 2015 to 2100 under the Shared Socioeconomic Pathways by using the logistic fitting model and iteratively identifying reference countries. Based on historical urbanization level database from the World Urbanization Prospects, projected urbanization levels and uncertainties are provided for 204 countries and areas every five years. The 2010–2100 year-by-year projected urbanization levels and uncertainties based on the annual historical data from the World Bank (WB) for 188 of countries and areas are also provided. The projections based on the two datasets were compared and the latter were validated using the historical values of the WB for the years 2010–2018. The updated dataset of urbanization level is relevant for understanding future socioeconomic development, its implications for climate change and policy planning.

## Abstract (summary)

**Table:** 

Measurement(s)	urbanization level
Technology Type(s)	model

## Background & Summary

Urbanization is a complex human-nature process. It changes the original nonurban areas to urban landscapes, and consequently alters the demographic, economic and social composition of the urban and rural areas^1,2^. Well-managed urbanization processes can help maximize the benefits of economic agglomeration while reducing environmental degradation and other potential adverse impacts^1,3^. Economies of scale and technological innovations in urban areas can promote economic growth and knowledge accumulation and create income and employment^4–6^ on the one hand, and reduce the per capita cost of providing infrastructure and social services^7^ on the other. Meanwhile, urbanization has exerted substantial negative impacts on the environment. For example, the growth rate of urban expansion is usually faster than that of the land protected as parks or reserves^8–10^; consequently, urban development often results in the extinction of native species, thereby threatening local ecosystems and ecological integrity.

Urbanization level is typically defined as a share of the population living in urban areas. Reliable projections of future urbanization dynamics and their uncertainties on a global scale thus can provide a solid basis for a broad range of studies. This includes future global socioeconomic development trajectories, climate change and its ecological and environmental implications^11–14^.

At the global scale, a widely used dataset for projecting the global urbanization level by 2050 is the national-scale five-year-interval World Urbanization Prospects (WUP) data released by the Population Division of the Department of Economic and Social Affairs of the United Nations (UNPD)^15^. Recently, Jiang and O’Neill further updated the estimates under the Shared Socioeconomic Pathways (SSPs)^16^. The two datasets are based on the UNPD’s urbanization level forecasting method (i.e., the difference in urban and rural population growth rates). However, this method contains several issues. First the previous global projections did not develop country-specific models to estimate urbanization level. In the WUP projections^17^, they established empirical linear relationships based on urbanization level and the difference in the growth rates of urban and rural population at several time points across 82 and 149 countries and areas, respectively. The estimated values based on such global uniform models may yield inaccurate results when compared to the historical urbanization level. Second, the coefficients in the established model and the identified reference countries for simulating future urbanization level were not updated in an iterative and dynamic fashion. For example, Jiang and O’Neill estimated the future urbanization level by adjusting the speed of urbanization derived from reference countries every 30 years^16^. However, urbanization is dynamic: some countries may change from developing to developed in less than 30 years. Accordingly, the coefficients and reference countries should be updated more frequently. Third, some projections used the logistic fitting model to estimate the urbanization level^18,19^. Such model assumes that a country’s urbanization level follows an S-shape curve and reaches a saturation stage (usually set to 80%) at which the urbanization level ceases to increase^20^. However, previous studies have found that a country’s urbanization level will continue to increase after reaching the saturation stage, especially in the context of globalization and global change^21^. Dynamically adjusting the saturation value to accurately simulate the changes in urbanization level after the saturation stage is thus a challenge. Fourth, previous studies did not provide a range of uncertainty. In long-term projections, the urbanization level is commonly used as an input in integrated assessment models or coupled earth system models. Therefore, its uncertainty can provide key information for the uncertainty and robustness of the results of these coupled models.

This study aims to update the global urbanization level from 2015 to 2100 under the SSPs, based on the previous estimations by Jiang & O’Neill^16^ using the logistic fitting modelling. We first establish a logistic model to fit the changes in urbanization level from 1950 to 2010, and then evaluate its performance. We provide two projections generated from two data sources (i.e., WUP 2018^15^ and World Bank (WB)^22^) with time steps of 5 years and 1 year, respectively. The two projections are compared, and the projected values based on the WB data 2010–2018 are validated with the historical values. We also include the uncertainty of the estimates at the national scale, which can be used as an input to other models. The updated dataset of urbanization level has a potential to be widely applied to the study of future socioeconomic development and climate change.

## Methods

**Fig. 1:**
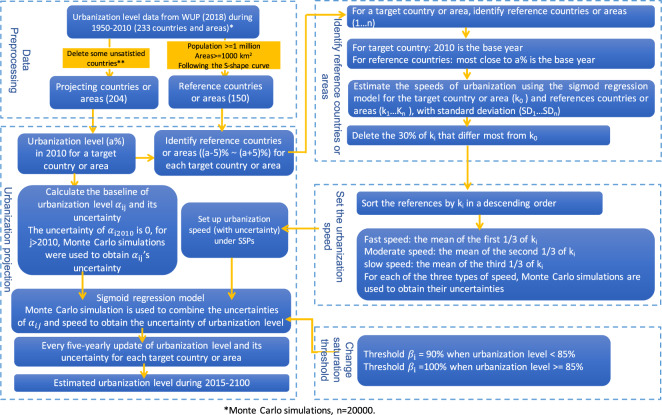
Technical flowchart for estimating global urbanization dynamics. *For statistical purposes, the data for China do not include Hong Kong and Macao, Special Administrative Regions (SAR) of China, and Taiwan Province of China. ** Countries and areas were eliminated because of incomplete data, or the S-shape curve not being followed, or the urbanization level in 2010 having reached 100%.

We estimated the dynamics of urbanization level for countries and areas based on the logistic fitting model outlined in the following four steps (Fig. 1). First, we pre-processed the data before projecting. Second, for each country and area where the level of urbanization is to be predicted, we selected eligible reference countries and areas for setting the urbanization speeds in the urbanization level simulations, and set the future urbanization development speeds in conjunction with the urbanization speed assumptions of the SSP storylines. Then, we set the upper limits of urbanization development, i.e. saturation value according to different urbanization levels. Finally, the previous steps were repeated every five years to simulate the dynamics of urbanization levels in the countries and areas of the world until the end of the century.

### Data pre-processing

We estimated the changes in urbanization level (i.e., the proportion of urban population to total population) based on two historical datasets, because the two datasets differ in temporal interval and the number of countries and areas. The first one is the five-year-interval dataset for 233 countries and areas from 1950–2010 from the World Urbanization Prospects: The 2018 Revision^15^. A total of 29 countries and areas were excluded from the analysis (Table 1). Two were excluded because of incomplete data. Meanwhile, 15 countries and areas were further excluded because their historical trends of urbanization level did not follow an S-shape curve, which is required by the logistic fitting model. In addition, 12 countries and areas with an urbanization level of 100% in 2010 were further excluded. Thus, a total of 204 countries were left for further estimation.

**Table 1:** Countries and areas excluded from the projections based on WUP 2018 and WB database.

Database	Reason for exclusion	Name	Code
World Urbanization Prospects: The 2018 Revision	Incomplete data	Tokelau; Wallis and Futuna Islands	TKL; WLF
	Not following a S-shape curve	Saint Helena; Tajikistan; Channel Islands; Isle of Man; Austria; Liechtenstein; Antigua and Barbuda; Aruba; Barbados; Caribbean Netherlands; Montserrat; Saint Lucia; Belize; Guyana; Micronesia (Fed. States of)	SHN; TJK; CHI; IMN; AUT; LIE; ATG; ABW; BRB; BES; MSR; LCA; BLZ; GUY; FSM
	With an urbanization level of 100% in 2010	China, Hong Kong SAR; China, Macao SAR; Singapore; Kuwait; Gibraltar; Holy See; Monaco; Anguilla; Cayman Islands; Sint Maarten; (Dutch part); Bermuda; Nauru	HKG; MAC; SGP; KWT;GIB; VAT; MCO; AIA; CYM; SXM; BMU; NRU
World Bank	Incomplete data	Tokelau; Wallis and Futuna Islands	XKX; SRB
	Not following a S-shape curve	Tajikistan; Channel Islands; Isle of Man; Austria; Liechtenstein; Antigua and Barbuda; Aruba; Barbados; Saint Lucia; Belize; Guyana; Micronesia (Fed. States of); Kyrgyz Republic; St. Kitts and Nevis; Samoa	TJK; CHI; IMN; AUT; LIE; ATG; ABW; BRB; LCA; BLZ; GUY; FSM;KGZ;KNA;WSM
	With an urbanization level of 100% in 2010	China, Hong Kong SAR; China, Macao SAR; Singapore; Kuwait; Gibraltar; Monaco; Cayman Islands; Sint Maarten, (Dutch part); Bermuda; Nauru	HKG; MAC; SGP; KWT; GIB; MCO; CYM; SXM; BMU; NRU

The second dataset is the annual urbanization level data from 1960 to 2019 for 215 countries estimated by the World Bank (WB)^22^. In the WB dataset, historical urbanization level data are collected and smoothed by the United Nations Population Division based on the World Urbanization Prospects: 2018 Revision as a data source^15^. Similarly, a total of 27 countries and areas were excluded in the data pre-processing step (Table 1), and a total of 188 countries and areas were projected for urbanization levels from 2010 to 2100 annually.

### Urbanization speed setting

Firstly, as the changes in urbanization level in a country or area may follow a similar path of another country or area that has passed the given urbanization level, we used the method proposed by Jiang and O’Neill^16^ and selected 150 countries and areas that could be used as reference countries and areas for estimating the varying speeds of urbanization level for each country and area (referred to as target country and area, Fig. 1). These reference countries and areas were required to meet the following criteria: 1) a population equal to or larger than one million^23^, 2) a territory equal to or larger than 10,000 km^2,^^24^, 3) historical trends of urbanization level follow an S-shape curve. Then, we selected a number of countries and areas every five years from these 150 reference countries and areas for a particular target country or area to estimate its urbanization speeds. The selected countries and areas should have an urbanization level similar to the target country and area (i.e., difference up to 5 percentage points) between 1950 and 2010. For example, if we want to estimate the urbanization level for China (target country) in 2015 and China’s urbanization level in 2010 was 49.2%, we would select countries and areas that had achieved an urbanization level between 44.2% and 54.2% during 1950 to 2010.

Secondly, we established country-specific logistic regression models using the historical data of the urbanization level of the target country or area (see **Establishing the fitting model** for details). Then, we further projected the trends of urbanization level using the varying speeds of urbanization level from the reference countries and areas. As the SSP storylines describe three kinds of urbanization speeds, i.e., fast, moderate and slow, we estimated three urbanization speeds with uncertainties for each target country. Specifically, we used the logistic regression model to simulate varying speeds and uncertainties (standard deviations) of the speeds of urbanization from the identified reference countries and areas for each target country or area. Following the method by Jiang & O’Neill^16^, we excluded 30% of the reference countries and areas whose speed of urbanization differ the most from the target country or area. For the remaining countries and areas, the mean of their urbanization growth rates in the top, middle and bottom 1/3 of the distribution were set as a fast, moderate and slow speed, respectively. Uncertainties for each of the fast, moderate and slow speed are simulated using Monte Carlo simulations (n = 20000). Then, the three levels of urbanization speed were used to simulate the varying trends of urbanization level under the SSPs (see **Simulating future urbanization level under different scenarios** for details). Fourth, we dynamically changed the saturation value of the logistic fitting model to solve the challenge of long-term simulation of urbanization level, especially for developing countries which are expected to become developed countries in the future (see **Changing the saturation value of urbanization** for details). Most importantly, the abovementioned three steps were iterated every five years, which means that the reference countries and areas, urbanization speeds and saturation threshold of urbanization level were adjusted every five years (Table 2).

**Table 2:** Urbanization speed differences among countries and areas under the Shared Socioeconomic Pathways (based on O’Neill *et al*.^28^).

	SSP1	SSP2	SSP3		SSP4		SSP5
Countries and areas	all	all	**Population**		**Income group**		all
			>10 million	<10 million	high- & middle-income	low-income	
Urbanization speed	moderate	slow	slow	stagnant	moderate	slow	fast

### Establishing the fitting model

The process of urbanization can be divided into several stages: the initial stage, the growth stage and the mature stage^20^. The growth stage can be further divided into two substages: the accelerating growth and decelerating growth substages. Previous studies have found that the S-shape curve can represent and fit these stages^21,25^. The logistic model used for simulating urbanization level is as follows:

1 $${Y}_{ij}=\frac{{\beta }_{ij}}{1+{e}^{{\alpha }_{ij}-{k}_{i}{t}_{j}}}$$

where ${Y}_{ij}$ represents the urbanization level of the targeted country *i* in Year *j*, and ${\beta }_{ij}$ represents the saturation value of the urbanization level, which is dynamically adjusted (see Section 3.2). In addition, ${\alpha }_{ij}$ refers to the baseline urbanization level of the targeted country *i* in Year *j*; ${k}_{i}$ reflects the urbanization speed of the targeted country; and ${t}_{j}$ is the difference between the simulated year and the base year.

${\alpha }_{ij}$ is calculated from ${Y}_{i}$ and ${\beta }_{i}$ at the base year. In 2010 (the first base year, our projections start from 2015), ${Y}_{i2010}$ has no uncertainty, so ${\alpha }_{i2015}$ has no uncertainty either. There is uncertainty in the predicted urbanization level ${Y}_{ij}$ for each five years after 2010, and the uncertainty is fed into ${\alpha }_{ij}$ using Monte Carlo simulation (n = 20000). Monte Carlo simulation is used to combine the uncertainties of ${\alpha }_{ij}$ and ${k}_{i}$ to obtain the uncertainty of urbanization level (n = 20000).

Previous studies have also shown that most countries and areas reach the maturity stage when their urbanization level reaches approximately 90%^21,25^. When a country or area enters the maturity stage, its urbanization level continues to increase but at a slower rate than that during the growth stage. In other words, it is necessary to adjust the saturation value of the urbanization level from 90% to 100% when a county or area enters the mature stage.

### Simulating future urbanization level under different scenarios

In 2010, the Intergovenmental Panel on Climate Change (IPCC) proposed the Shared Socioeconomic Pathways (SSPs) storylines which describe a variety of social and economic development paths in the 21^st^ century both qualitatively and quantitatively. These paths were then combined with various climate change trends to form a framework of scenarios for climate change mitigation, adaptation and impact^26,27^. The SSP storylines specify alternative paths covering a variety of socioeconomic factors, including population, urbanization level, education, economic growth, social equity, policy orientation, institutional efficiency, science and technology, and environment and natural resources. Following the SSP storylines^28^, we assigned fast, moderate, slow or stagnant urbanization speed for each countries or areas under the different SSPs (Table 2). In SSP1 (sustainability), it is assumed that the world gradually but pervasively evolves toward a more sustainable route while respecting perceived environmental constraints; thus, the increase in urbanization level will maintain a moderate speed for all countries and areas. Under SSP2 (middle of the road), the world is moving down a path where social and economic tendencies do not deviate significantly from past patterns; therefore all countries and areas follow a slow process of urbanization. Under SSP3 (regional rivalry), policies are oriented towards regional security with a resurgent nationalism, many countries are striving to sustain living standards including achieving energy and food security goals. In this circumstance, we assume that countries and areas with populations over ten million are seeing a slow speed of urbanization, while those with populations less than ten million are expected to remain stagnant because a country or area’s urbanization level is more likely to be affected by natural disasters, conflicts, and economic recessions when it has a smaller population size (see Supplementary Information for details). Under SSP4 (inequality), economic growth and social development are highly unequal across regions, assuming slow growth for low-income groups and medium growth for others. For this pathway, urbanization speed is moderate for high- and middle-income economies but slow for low-income economies. Under SSP5 (fossil-fueled development) path, economic development is the ultimate goal and is highly dependent on fossil fuel consumption. For this pathway, the urban area is better managed, but some sprawl occurs over time; hence, all countries and areas follow a fast process of urbanization.

This study includes two projections based on WUP 2018 and WB datasets, respectively. We set urbanization speeds under the five SSP scenarios (Table 2) for both projections. For the SSP4 scenario, since urbanization speed varies among different income levels, we need to select the income level for each country or area first, and then determine whether it follows a moderate or fast speed (Table 2).

For 204 countries and areas with the data obtained from the WUP 2018, we further divided 194 of these countries and areas into three groups by their income level in 2020, i.e., high-, middle- and low-income economies according to a division by the World Bank^29,30^. We set the SSP4 scenario for these 194 countries and areas because only 194 of our 204 countries and areas are included in the data on economies from the World Bank (see Suppl. Table 1). In the current fiscal year of 2020, a low-income economy is defined as an economy with a 2018 gross national income (GNI) of less than or equal to $1,025. A lower middle-income economy is defined as the one with a GNI between $1,026 and $3,995, while an upper-middle income economy is defined as the one with a GNI between $3,996 and $12,375. A high-income economy is an economy with a GNI higher than $12,375.

As for the projections based on the WB dataset, we further divided all 188 of these countries and areas into three groups by their income level in 2020. The projection procedure is exactly as described above, except that the selection of reference countries and areas for the projection is done annually instead of every 5 years. All 188 countries and areas are included in the World Bank’s data on economies (see Suppl. Table 2), so the SSP4 scenario is set for each of these 188 countries and areas.

### Changing the saturation value of urbanization

As the time period of the projections reaches 90 years, some countries and areas may change from developing to developed economies. Thus, the saturation values of the urbanization level should be dynamically adjusted. Here, we followed the method proposed by Chen *et al*.^19^ to adjust the saturation values. For countries with urbanization levels lower than 85%, the saturation value of the urbanization level used in the logistic function was set to 90%. For countries with urbanization levels exceeding 85%, the saturation value was dynamically set to 100%. This process was applied to each target country or area every five years (WUP 2018 based projections) or annually (WB based projections).

## Data Records

The projections are available at the public repository Figshare^31^. The data projected based on the WUP 2018 database are stored under the ‘WUP 2018’ folder, and the data predicted based on the WB database are stored under the ‘WB’ folder. The urbanization level and uncertainty for each country and area are stored in ‘.xls’ files named starting with different ‘SSPs’, and the files ending with ‘SD’ represent the standard deviation of the projections.

In ‘WUP 2018’ folder and ‘WB’ folder, we also provided files named ‘the 100% urbanization level countries and areas’ contains countries and areas in which urbanization levels were 100% in 2010 or 2009 and assumed their urbanization levels will stay 100% in the future.

## Technical Validation

First, we calibrated the logistic regression model using historical values of WUP 2018 from 1950 to 2005. The root-mean-square error (RMSE) was used to evaluate the performance of the logistic regression model. Based on the historical data and the logistic regression model, the urbanization levels of 204 countries and areas from are simulated, and the RMSE between the real value and the simulated value is calculated for each country or area. Approximately 87% of the countries and areas have a RMSE less than 4.85%, and only one has a RMSE greater than 10% (Table 3).

**Table 3:** The distribution of the root mean squared errors between the simulated and historical values of urbanization level of WUP 2018 dataset and WB dataset.

**WUP 2018 dataset**	RMSE(%)	0.36–2.60	2.60–4.85	4.85–7.09	7.09–9.33	9.33–11.85
	numbers of countries	102	75	21	5	1
**WB dataset**	RMSE(%)	0.17–1.54	1.54–2.90	2.90–4.27	4.27–5.64	5.64–7.01
	numbers of countries	75	57	34	14	8

Similarly, we calibrated the logistic regression model based on historical urbanization level data from WB in 2010. The urbanization levels of 188 countries and areas from 1960–2009 are used for model calibration, and the RMSE between the real value and the simulated value is calculated for each country or area. Approximately 88% of the countries and areas have a RMSE less than 4.27%, and all countries and areas has a RMSE less than 10% (Table 3).

**Fig. 2:**
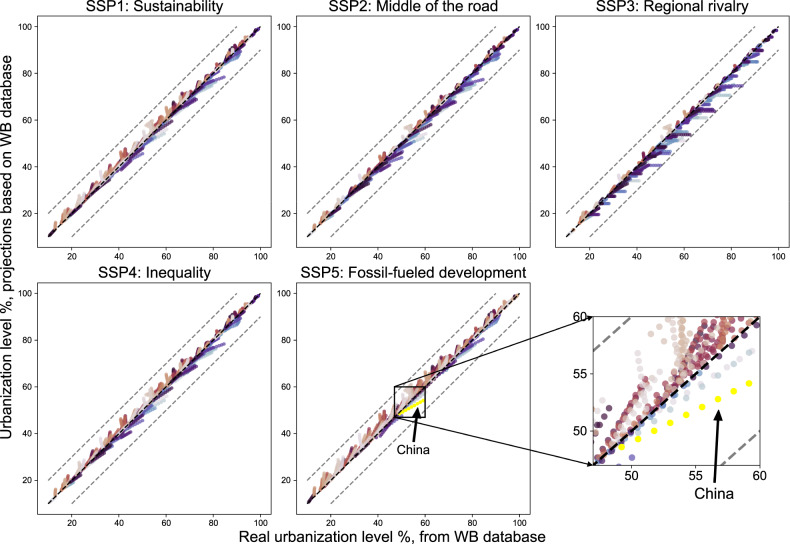
Comparisons between the simulated urbanization level (based on WB database) and historical values from 2010 to 2018 under different SSP scenarios. The gray dashed line representing a 10% gap of the 1:1 diagonal line. In each subplot, one array of points with the same color represents the changes in urbanization level from 2010 to 2018 for one country or area.

Second, we used the calibrated model to conduct model validation with historical data. We compared the estimated urbanization level during 2010–2018 based on the historical WB dataset from 1960–2009 against annual historical data from the WB dataset among 188 countries and areas. The difference between the projected results and historical values is less than 10% for all countries. The projected values tend to be higher than the real values in the SSP1 and SSP5 scenarios, i.e. the sustainability and fossil-fueled development paths, and significantly lower than the real values in the SSP3 scenario, i.e. the regional rivalry (Fig. 2).

**Fig. 3:**
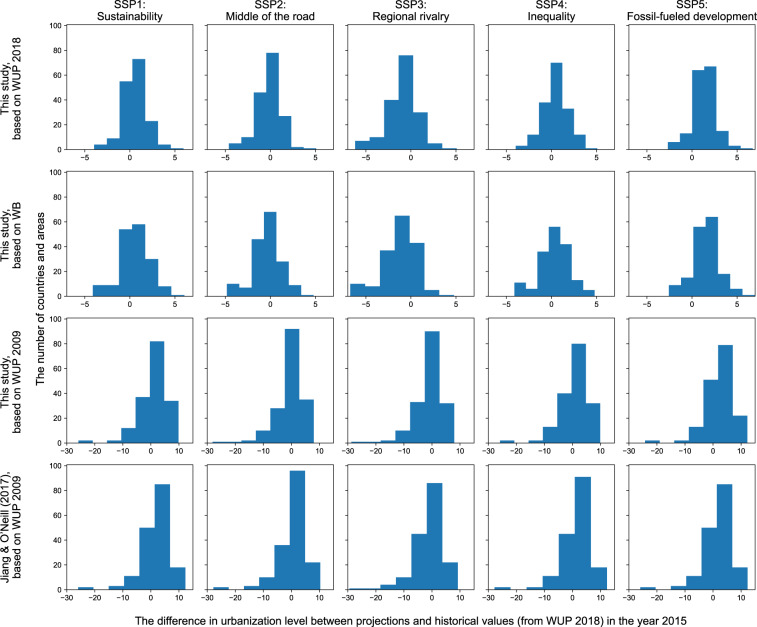
Comparisons between different projections and historical urbanization level values in 2015 under different SSP scenarios. The rows in this panel represent the difference between this study’s simulated urbanization level (based on WUP 2018, WB, and WUP 2009 databases), Jiang & O’Neil’s^16^ simulated urbanization level (based on WUP 2009 database) and historical values in 2015 under different SSP scenarios, respectively. A total of 169 overlapping countries and areas are compared.

Third, we further compared the estimated urbanization level (using our methods, based on the WUP 2018, the WB and the WUP 2009^32^, respectively) in 2015 against historical value in 2015 (from the WUP 2018^15^) and the previous estimation from Jiang and O’Neill^16^ (Fig. 3). Only 169 countries and areas that overlap among the four sets of projections, and the urbanization level in 2015 from WUP 2018^15^ are compared.

Using the methodology of this paper and WUP 2018 dataset, the absolute difference between the projections based on the WUP and WB databases and the historical values for 2015 range from −6% to 7%. By contrast, the absolute values of the differences between Jiang & O’Neill’s projections^16^ based on WUP 2009^32^ and the 2015 historical values^15^ can reach up to more than 20%. For instance, Japan (JPN) and Equatorial Guinea (GNQ) have large deviations from historical values in all four scenarios, with absolute differences from 21% to 29%.

The projection made by Jiang and O’Neill^16^ was based on the WUP 2009 revision^32^. Thus, we performed another urbanization projection based on the WUP 2009 revision^32^ using our method, to compare the previous projection made by Jiang and O’Neill^16^ on the same basis (Fig. 3, row 3 & 4). Using the methodology of this paper, the range of absolute difference between the projections based on the WUP 2009 revision and the historical values for 2015 (−28.7%~12.2%) is nevertheless smaller than that of Jiang and O’Neill’s projections (−29.4%~12.3%).

The new dataset thus has provided comparable projections including uncertainty estimates for more than 180 countries and areas. The number of countries and areas included in the three projections are as follows: our projection based on the WUP 2018^15^ (204 countries); our projection based on WB^22^ (188 countries); and Jiang & O’Neill’s projection^16^ (193 countries). The countries that do not overlap are shown in Table 4.

**Table 4:** Countries and areas in the three projections that do not overlap with each other.

	In this article (based on WUP 2018), not in Jiang & O’Neill^16^ (based on WUP 2018)	In Jiang & O’Neill^16^ (based on WUP 2018), not in this article (based on WUP 2018)	In this article (based on WB), not in Jiang & O’Neill^16^ (based on WUP 2009)	In Jiang & O’Neill^16^ (based on WUP 2009), not in this article (based on WB)
countries and areas	SYC; SSD; ESH; FRO; AND; SMR; VGB; CUW; DMA; KNA; TCA; FLK; GRL; SPM; KIR; MHL; MNP; PLW; ASM; COK; NIU; TUV; Taiwan (province of CHN)	AUT; PSE; TJK; ABW; FSM; LCA; BRB; BLZ; GUY; KWT; SGP; HKG; MAC	AND; ASM; CUW; DMA; FRO; GRL; KIR; MHL; MNP; PLW; SMR; SSD; SYC; TCA; TUV; VGB	AUT; GZ; PSE; SRB; TJK; ABW; FSM; LCA; WSM; MYT; GUF; BRB; BLZ; MTQ; GLP; GUY; REU; KWT; SGP; HKG; MAC

For KWT, SGP, HKG, MAC, the urbanization levels of these four countries and areas has reached 100% in 2010, so no prediction is made in this study.

**Fig. 4:**
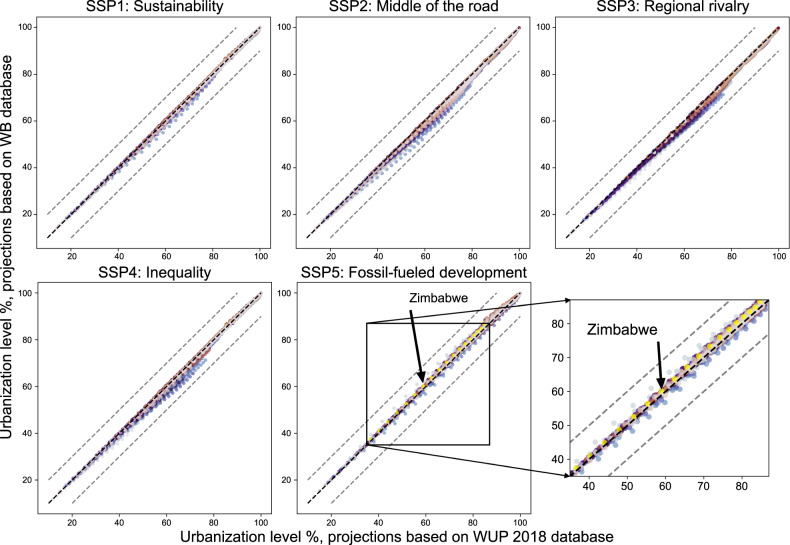
Comparisons between the simulated urbanization level (based on WB database) and the simulated urbanization level (based on WUP 2018 database) from 2015 to 2100 (every five years) under different SSP scenarios. The gray dashed line representing a 10% gap of the 1:1 diagonal line. In each subplot, one color represents one country or area for the urbanization levels from 2015 to 2100 (every five years).

Fourth, we compared the differences between our two projections based on WUP 2018 and WB, respectively. Since the two datasets have different time interval, we compared the overlapped years (i.e., every five years) from 2015 to 2100 (Fig. 4). The comparison shows that the differences between the two projections for each scenario are all within the range of 10%. In other words, the two projections based on the two data sources with different temporal resolution of urbanization level would yield similar results.

Finally, in addition to comparing the differences between estimated values and historical values for individual country or area, we also compared the difference at the global scale. Using our urbanization level projections with the 2015 population data provided by the WUP^33^, we first calculated the urban population for each country and area separately under different scenarios. Then we combined the total and urban population of these countries and areas. Finally, dividing the urban population by the total population of the world yields the expected global urbanization level for each scenario. The results (Table 5) show that the absolute differences between our WUP-based predictions and the true values are less than 1% at the global scale, and the absolute differences between the WB-based predictions and the true values are less than 1.3% in 2015. This also demonstrates that our study reliably portrays the level of urbanization at the global.

**Table 5:** The absolute differences between the simulated and historical values of urbanization level of WUP 2018 dataset and WB dataset (all countries and areas are merged into one whole world).

	WUP 2018 dataset			WB dataset		
	Urbanization level in 2015	Absolute difference from the real value	Merged countries and areas	Urbanization level in 2015	Absolute difference from the real value	Merged countries and areas
**SSP1**	53.72%	−0.13%	204	53.52%	−0.25%	188
**SSP2**	52.99%	−0.86%	204	52.53%	−1.23%	188
**SSP3**	52.93%	−0.92%	204	52.47%	−1.30%	188
**SSP4**	53.64%	−0.21%	194	53.43%	−0.33%	188
**SSP5**	54.53%	0.68%	204	54.62%	0.86%	188
**Real**	53.85%	0	204	53.76%	0	188

## Usage Notes

**Fig. 5:**
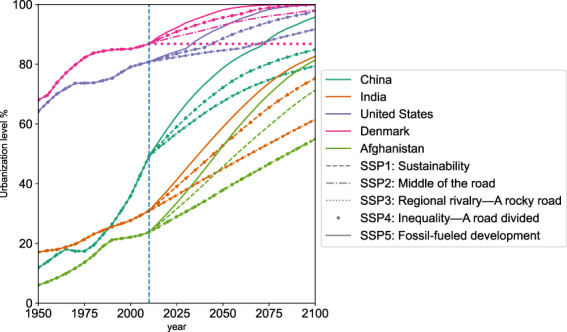
Urbanization levels of the five example countries. For clarity of expression, we did not delineate the standard deviation but provided them in the ‘_SD.xls’ files^31^.

**Fig. 6:**
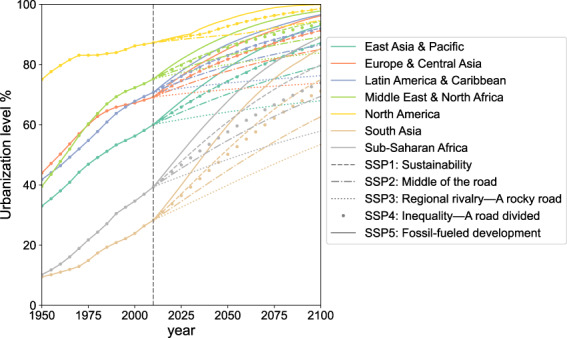
Urbanization levels of seven regions under different urbanization speed. The lines are the average values for different regions. For clarity of expression, we did not delineate the standard deviation but provided them in the ‘_SD.xls’ files^31^.

Our projection can show the urbanization development path for each country or areas in this century under the different SSP scenarios (Fig. 5). In addition to the absolute value of urbanization level from the previous projections^16^, we also included the uncertainties for each country^31^. The trend of urbanization level for each region as a whole can also be aggregated (Fig. 6).

## Supplementary information

Supplementary Information

Suppl. Table 1

Suppl. Table 2

## Data Availability

All python codes (python 3.9.6, https://www.python.org) for creating urbanization level projections are stored in public repository Figshare^31^.
