# Supplementary material for: Updating global urbanization projections under the Shared Socioeconomic Pathways
Source: Sci Data. 2022 Mar 31;9:137. doi: 10.1038/s41597-022-01209-5 (PMC8971473; doi:10.1038/s41597-022-01209-5)
Supplement: Supplementary file 1 — Supplementary Information [file 41597_2022_1209_MOESM1_ESM.docx]

### Supplementary Text

For the SSP3 (regional rivalry) scenario, we assumed the urbanization speeds vary across countries and areas with different population sizes. We firstly checked those countries and areas with urbanization progress not following a S-shape curve based on WUP dataset (Table 1, Supplementary Fig. 1), and found that these countries and areas often follow a declining or stagnant urbanization process. And these countries and areas all have a population size under 10 million (Supplementary Fig. 1). Although no significant relationship was found between the level of urbanization and population (Supplementary Fig. 2), we conjecture that countries and areas with smaller population sizes are more vulnerable to emergencies, conflicts, or recessions. For example, the global recessions around 1975, 1982, and 1991^1^ may have led to a reverse urbanization process in most of the countries and areas in Supplementary Fig. 1. In addition, the following events may also have affected the urbanization process in these countries: the dissolution of the West Indies Federation in 1962, the riots of protesting against new taxes and the hit of Hurricane Luis in 1995 for Antigua and Barbuda^2^; the 1995 and 1997 volcano eruptions for Montserrat^3^; the violence and anti-Russian riots in 1970s and the collapse of the Soviet Union in 1991 for Tajikistan^4^. The SSP3 scenario is exactly a rocky road with resurgent nationalism, concerns about competitiveness and security, and regional conflicts, forcing many countries to strive to maintain living standards^5^. Thus, we assigned slow urbanization speed for the countries and areas with populations over ten million, stagnant urbanization for those with populations less than ten million. All these population and urbanization level data are from the WUP 2018 revision^6^.


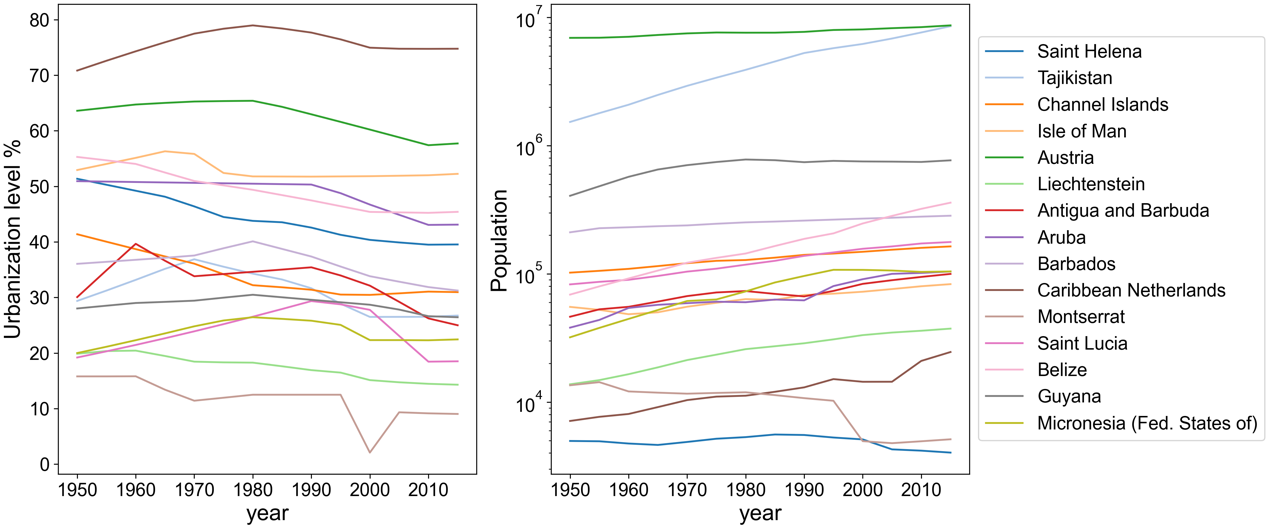


Supplementary Fig. 1 The urbanization level and population of non-S-shape curve countries and areas^6^.


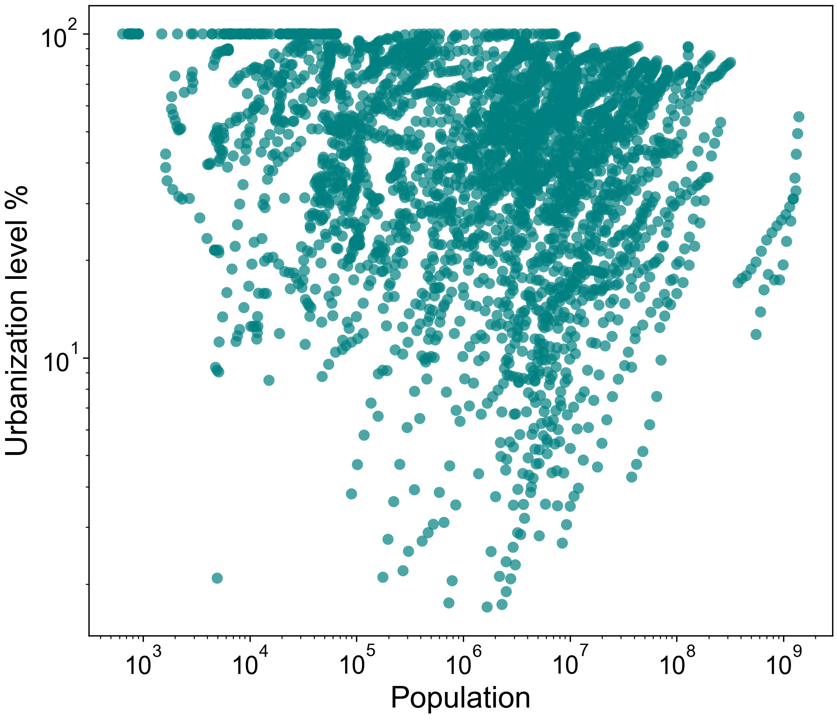


Supplementary Fig. 2 The relationship between the urbanization level and the population of 233 countries and areas in the world, from 1950-2015^6^.

1. International Monetary Fund. *World Economic Outlook: Crisis and Recovery.* (Washington, DC : International Monetary Fund, 2009). <https://www.imf.org/en/Publications/WEO/Issues/2016/12/31/Crisis-and-Recovery>
2. The BBC. *Antigua and Barbuda profile – Timeline.* <https://www.bbc.com/news/world-latin-america-18707512> (2021).
3. The BBC. *Tajikistan profile – Timeline.* <https://www.bbc.com/news/world-asia-16201087> (2021).
4. The BBC. *Montserrat profile – Timeline.* <https://www.bbc.com/news/world-latin-america-20257216> (2021).
5. O’Neill, B. C. et al. The roads ahead: Narratives for shared socioeconomic pathways describing world futures in the 21st century. *Global Environmental Change* **42**, 169–180 (2017).
6. UNPD (United Nations, Department of Economic and Social Affairs, Population Division). World Urbanization Prospects: The 2018 Revision, Online Edition. <https://population.un.org/wup/Download/> (2018).
